# Analysis of the effectiveness of cinnamon (*Cinnamomum verum*) in the reduction of glycemic and lipidic levels of adults with type 2 diabetes: A study protocol

**DOI:** 10.1097/MD.0000000000018553

**Published:** 2020-01-03

**Authors:** José Claudio Garcia Lira Neto, Marta Maria Coelho Damasceno, Márcia Aparecida Ciol, Roberto Wagner Júnior Freire de Freitas, Márcio Flávio Moura de Araújo, Carla Regina de Souza Teixeira, Gerdane Celene Nunes Carvalho, Kenya Waléria de Siqueira Coelho Lisboa, Danilo Ferreira de Souza, Jéssica de Menezes Nogueira, Regina Lúcia Lino Marques, Ana Maria Parente Garcia Alencar

**Affiliations:** a Department of Nursing, Federal University of Ceará, Fortaleza, Ceará, Brazil; b Department of Rehabilitation Medicine, University of Washington, Seattle, Washington; c Department of Family Health, Oswaldo Cruz Foundation, Eusébio, Ceará; d Department of Nursing, São Paulo University, Ribeirão Preto, São Paulo; e Department of Nursing, Piauí State University, Picos, Piauí; f Department of Nursing, Cariri Regional University, Crato; g Department of Nursing, Juazeiro do Norte College, Juazeiro do Norte, Ceará; h Department of Nursing, Federal University of Piauí, Floriano, Piauí; i Department of Nursing Hematology and Hemotherapy Center of Ceará, Fortaleza, Ceará, Brazil.

**Keywords:** cinnamon, herbal medicine, type 2 diabetes mellitus

## Abstract

**Background:**

Type 2 Diabetes Mellitus (T2DM) is a chronic disease that is increasing the number of cases worldwide. The treatments currently used have not worked as expected. Alternative and complementary medicines were inserted in health services, especially in primary care, as an attempt to minimize risks and help control diseases such as diabetes. Among the herbal medicines used stands out cinnamon, which can serve as an adjuvant in the control of diabetes.

**Objective:**

To analyze the effectiveness of 3 grams of cinnamon (*Cinnamomum verum*) per day for 90 days in reducing glycemic and lipid levels in adults with T2DM compared with placebo

**Methods:**

A randomized, double-blind, placebo-controlled, phase II trial, which will be conducted at basic health units in the city of Parnaíba, state of Piauí, Brazil. In total, 130 people diagnosed with T2DM, followed at health units, with hemoglobin A1c > 6.5%, and using oral antidiabetic medicines, are expected to participate in the study. The intervention will last for 3 months, and each participant will receive a total of 3 bottles containing 120 capsules in each bottle of cinnamon or placebo. Each person should take 4 capsules daily, for 90 days. The patients will be distributed into the 2 groups by performing block randomization (n = 6) at a ratio of 1:1 according to a code generated by a software. Assessments of socioeconomic, clinical, lifestyle, anthropometric, and laboratory variables will be performed in 2 separate visits.

**Discussion:**

This study will be the first to investigate cinnamon to reduce glycemic, lipid, and anthropometric levels in Brazil. In case of favorable results, this therapy may be used as an alternative or additional medicine in cases where only oral antidiabetic agents are used and can promote the use of the product to minimize future complications of patients with diabetes and people who do not have the disease.

**Trial registration:**

RBR-2KKB6D, registered on December 11th, 2018.

## 1 Introduction

Type 2 diabetes mellitus (T2DM) is a metabolic disorder known as a global health problem, characterized by elevated blood glucose levels. It is estimated that nearly 200 million people with diabetes are undiagnosed and; therefore, at greater risk of developing complications including kidney failure, blindness, amputations, heart disease, and stroke. These complications increase the cost of healthcare and decrease the quality of life.^[1]^

According to the International Diabetes Federation, the general target for glucose control in T2DM should be less than 7% of hemoglobin A1c (HbA1C) (53 mmol/mol).^[1]^ The T2DM management can prevent or reduce the risk of such complications. A 1% reduction in HbA1C is reported to be associated with a 21% lower risk of diabetes complications.^[2]^ Strategies to improve glycemic status are worth investigating. Losing weight, decreasing lipids, anthropometric indices, (eg, waist circumference) and body composition, along with lifestyle changes and drug therapy, are the main treatment for T2DM.^[3–5]^

However, due to the high prices of prescribed medications, polypharmacy, inadequate diet, the degree of dementia in some cases, low economic power, or even insufficient education, patients end up in clinical inertia.^[2]^

In this direction, in Brazil, the National Policy of Integrative and Complementary Practices was created in 2006,^[6]^ with the objective of implementing alternative treatments to evidence-based medicine in the Brazilian public health network, through the Unified Health System. The tendency of alternative and complementary therapies is increasing considerably, particularly herbal medicine, around the world.^[5]^ However, in many cases, there is insufficient evidence about its safety and efficacy.

Among the many herbal medicines examined so far,^[7–9]^ one deserves special mention for being useful in reducing both glycemic and lipid (and even anthropometric) biomarkers in patients with diabetes, which is the cinnamon.^[5]^ Despite encouraging results, the use of this product has not yet been investigated in countries such as Brazil, where the population has unique life characteristics, and where primary care health care has not been sufficient to manage diabetes patients with quality.

## 2 Justification

The interest of this project is the evaluation of the efficacy of cinnamon to control the glycemic and lipid levels of people with T2DM. The justification for the importance of the proposal considers recent data from research conducted in Brazil. Among other information, it was revealed that the costs attributed to adult hospitalizations for diabetes were 19% higher than hospitalizations for other diseases. In addition, hospitalizations for cardiovascular diseases related to diabetes accounted for 45%.^[10]^ Certainly, this scenario is closely related to the quality of disease control and, therefore, requires, among others, the discovery of new adjuvant technologies for this control.

The implementation and applicability of the proposal is supported by the pillars of the Brazilian health system, and undoubtedly intends to promote its use as an adjuvant alternative in T2DM control, and may be made available by the Ministry of Health, aiming at reducing the economic expenses on health services. Also, this product may contribute to the minimization of pathogenesis-related adversities of high glycemic and lipid values in people who do not yet have chronic diseases. If cinnamon is effective in controlling T2DM, it will allow better resource allocations to manage this disease, producing health-promoting effects on costs and budgeting for other needs.

## 3 Objectives

### 3.1 General objective

To analyze the effectiveness of 3 grams of cinnamon (*Cinnamomum verum*) per day for 90 days in reducing glycemic and lipid levels in adults with T2DM compared with placebo.

### 3.2 Specific objectives

(1) To investigate the efficacy of cinnamon in reducing the glycemic levels (HbA1C and fasting venous glucose) of people with T2DM compared with placebo;
(2) To analyze the efficacy of cinnamon in lowering lipid levels (total cholesterol, low density lipid-cholesterol and triglycerides), and increasing high density lipid-cholesterol in people with T2DM compared with placebo;
(3) To evaluate the efficacy of shin in reducing anthropometric values (weight, waist circumference, waist circumference, neck circumference, thigh circumference, hip circumference, body mass index, central adiposity index, neck-thigh ratio, waist ratio -quadrille, waist-to-thigh ratio, waist-to-height ratio) in people with T2DM compared with placebo.

## 4 Methods

### 4.1 Trial design

A randomized, double-blind, placebo-controlled, phase II trial, which will be conducted at Basic Health Units in the city of Parnaíba, state of Piauí, Brazil. In total, 130 people diagnosed with T2DM, followed at health units, and using oral antidiabetic medicines, are expected to participate in the study – according to sample calculations. This study complies with ethical guidelines in humans. The study was approved by the Research Ethics Committee of the State University of Piauí, under Opinion number 3.447.415, and was registered with the Brazilian Clinical Trials Network/World Health Organization Primary Registries under protocol RBR-2KKB6D.

For the purpose of greater transparency and quality of the research, Table 1 summarizes the enrolment, intervention, and assessment schedule, all of which are in accordance with the standard protocol items: recommendations for interventional trials recommendations.

**Table 1:**
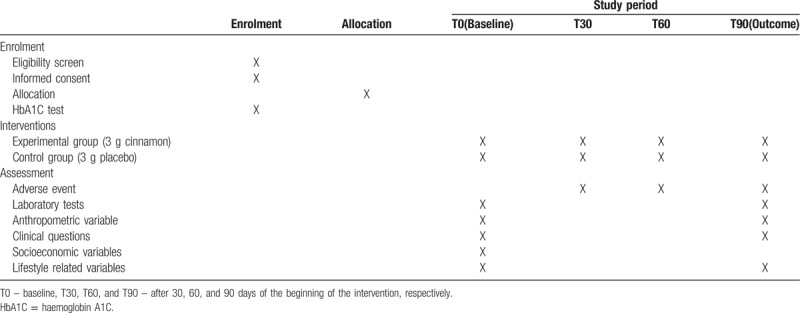
Schedule of enrolment, intervention, and assessment of treatments.

#### 4.1.1 Baseline and post-intervention variables

##### 4.1.1.1 Socioeconomic variables (baseline only)

Will be investigated: family economic income, years of study, marital status, type of work, skin color, gender, and so on.

##### 4.1.1.2 Clinical questions

Will be investigated: how long the patient has diabetes, what medications the patient takes, if have some allergies, where the patients get the medicines, how often do the patients see the doctor, which professional does direct follow-up and patient health management, if they use alternative and complementary practices, if any herbal medicine is used for any clinical condition, etc. The blood pressure will be measured.

##### 4.1.1.3 Lifestyle related variables

Will be investigated: If the patient uses alcohol, tobacco, if they do some physical activity and how often, how is the diet, and so on.

##### 4.1.1.4 Anthropometric variables

Will be investigated: waist circumference, thigh circumference, neck circumference, abdominal circumference, hip circumference, height, weight, body mass index, central adiposity index, waist-hip ratio, neck-thigh ratio, and so on.

##### 4.1.1.5 Laboratory tests

The tests will be collected: fasting glucose, HbA1C, total cholesterol, HDL, LDL, triglycerides, and the homeostatic model assessment of insulin resistance (HOMA-IR) index will be calculated.

#### 4.1.2 Outcomes

##### 4.1.2.1 Primary outcome

Reduction of glycemic levels (HbA1c and fasting venous glucose) after 3 months of use of the received capsules (cinnamon or placebo).

##### 4.1.2.2 Secondary outcome

After 3 months of use of the received capsules (cinnamon or placebo), the following secondary outcomes will be collected:

(1) Reduction of lipid levels (LDL cholesterol, triglycerides and total cholesterol);
(2) Increase HDL cholesterol levels.
(3) Reduction of anthropometric measurements (weight, waist circumference, waist circumference, neck circumference, thigh circumference, hip circumference, body mass index, central adiposity index, neck-thigh ratio, waist-hip ratio, waist-thigh ratio, waist-to-height ratio).

### 4.2 Participants

People diagnosed with T2DM, from Parnaíba city, possibly eligible to participate in the study. Recruitment will be based on the list of people with T2DM, registered and followed up at each Health Unit. Recruitment will be at least 10 days before the start of data collection. Recruitment will be carried out by community health workers from each health unit.

#### 4.2.1 Inclusion criteria

(1) Men or women aged 18 to 80 at Visit #1;
(2) Having a diagnosis of T2DM, confirmed for at least 5 years;
(3) On oral antidiabetic drugs;
(4) HbA1C > 6.5% (Tests performed before the subjects entered the research);
(5) Preserve cognitive functions, according to the Mental State Mini-Exam.

#### 4.2.2 Exclusion criteria

(1) Patients with cinnamon allergy;
(2) Patients using cinnamon or another herbal medicine for diabetes treatment;
(3) Patients diagnosed with alcoholic liver disease, cirrhosis or abnormal basal liver function;
(4) Patients on insulin therapy;
(5) Women who are breastfeeding, pregnant or taking contraceptives;
(6) Patients with heart, liver or respiratory failure;
(7) Patients with bleeding disorders who are taking coumarin derivatives (eg warfarin).

#### 4.2.3 Discontinuing criteria

(1) A participant wishing to withdraw from the study for any reason;
(2) Serious adverse events or unusual changes in laboratory tests;
(3) Patients taking another type of herbal medicine during this study.

### 4.3 Sample size

The sample will be calculated based on a previous literature study.^[11]^ The sample size observed in the Vafa's^[11]^ study was calculated on the difference in HbA1C (%) for each person between the initial and final values, resulting in: {mean of differences in the cinnamon group – mean of differences in the control group} / {2 [((SD cinnamon group) 2 + (SD control group 2)/2]} = { –0.44 –(–0.10)}/{2 [(0.722 + 0.652)/2]} = 0.4957 ≅ 0.50. So, if we consider the effect of 0.50, we need 64 participants per group (128 total) for 80% power. Although we do not expect a large loss, if we assume a 20% loss, we will need to recruit about 162 participants to have 130 participants at the end of the study. The calculations were made in GPower 3.0 by choosing the *t* test for independent samples.

### 4.4 Randomization

The patients will be distributed into the 2 groups by performing block randomization (n = 6) at a ratio of 1:1 according to a code generated by the statistical package for the social sciences (SPSS) software version 20.0 for Mac (IBM Corporation, Armonk, NY).

#### 4.4.1 Allocation

A number will be assigned to each patient, and this number will be labeled on the 3 bottles the patient receives. After this step, the member of the research group returns the box containing all the bottles to the main researcher. When the principal investigator or researchers involved in data collection deliver the vials to the patients, the number contained on the vial will be recorded next to each patient's name.

The registration of the numbers for each patient will be done in written and electronic form. The printed records will be stored in a folder containing the study participants’ documents. Electronic records will be entered into an excel spreadsheet. A copy of the written records will be sent to the study supervisor/coordinator. Each patient's identification number will be listed and registered by the member. This way, the member will know which group each participant will be in.

The list of numbers assigned to each patient will be printed and stored in a sealed envelope, under the responsibility of the member. A copy will be placed in another sealed envelope and will be under the responsibility of the study supervisor/coordinator.

The researcher will also record the data in an excel spreadsheet, save it to a personal computer and include the records in an online “cloud,” with login and password under the researcher's possession only. The lead researcher will only know to which group each participant was assigned at the end of the intervention period.

### 4.5 Interventions and procedures

Participants will be allocated into 2 different groups (experimental group and control group). The intervention will last for 3 months, and each participant will receive a total of 3 bottles containing 120 capsules in each bottle of cinnamon or placebo. Each person should take 2 capsules with water 30 minutes before breakfast and 2 capsules 30 minutes before lunch daily for 90 days. A new bottle containing ginger or placebo capsules was delivered at intervals of 25 to 29 days. Participants will be assigned to the experimental (cinnamon) or control (placebo) group at random. Assessments of socioeconomic, clinical, lifestyle, anthropometric, and laboratory variables will be performed in 2 separate visits, like described in Table 1.

(1) Experimental treatment - Cinnamon capsules (*C verum*), at a dose of 3 g/d, and oral antidiabetics.
(2) Control treatment – Microcrystalline cellulose capsules (placebo), at a dose of 3 g/d, and oral antidiabetics.

All patients will be encouraged to continue taking their medications routinely, following the recommendations of their attending physicians. Participants will be instructed on how to take the capsules. Both cinnamon and placebo will be encapsulated and packaged in identical vials, containing a label with information on the dosage, expiration date of the product (longer than the intervention period) and the return date. Each vial will be numbered to facilitate participants’ randomization process. To promote participant retention, phone calls will be applied, weekly.

Patients will be encouraged not to change their physical activity or eating routine during the data collection period. All participants will be informed of the risks and benefits of the study and will be aware that they may leave the study at any time for any reason.

After recruitment, evaluation of participation criteria and acceptance of participants to be part of the study, the researchers will set a date to begin data collection. The collection will be divided into 2 steps. In the first stage, participants will be instructed about the study and will collect all socioeconomic, clinical, and laboratory variables. One of the participation criteria will be conditioned to the values of HbA1C. Once the test results are in hand, the principal investigator will schedule a second time with patients in the follow-up health units to be randomized to receive the vials containing the cinnamon or placebo capsules. To evaluate adherence, Morisky test will be applied.

All treatments will be performed by a group of nurses who will undergo a training and calibration exercise in the initial phase of the study. The major researcher will perform the intervention and data collection and will be blind about which group it will conduct.

Blood samples (10 mL) will be taken after 10 to 12 hours of fasting. Samples will be centrifuged at room temperature at 3000 rpm for 10 minutes to separate serum from blood cells. The tests will be determined by the enzymatic colorimetric method with commercially available kits (Pars Azmun Co., Tehran, Iran) on an automated analyzer (Abbott, model Alcyon 300, Abbott Park, IL). In turn, for HOMA-IR, it will be calculated by multiplying the glucose by insulin (μUI/mL), both fasting, and dividing by 22.5, the cutoff established was 2.5.

Venipuncture, manipulation, and analysis of biological samples will be performed by trained professionals and the analysis will be conducted in a CONTROLLAB quality seal clinical analysis laboratory, intermediated by the Brazilian Society of Clinical Pathology and Laboratory Medicine, and the Program quality seal, National Quality Control.

Blood tests results will be delivered to participants after the full study review is completed so that participants do not jump to conclusions about the intervention as well so that they do not start using cinnamon (or not) without awareness of the true efficacy. Between visits, the lead researcher will call participants once every 15 days to follow up on the study. There will be no instruments to complete during these phone calls, but the researcher should record information about the call and related adverse events.

The investigator will analyze, through discussion with the patient, the occurrence of adverse events during each visit, and record the information. Adverse events will be recorded with each participant's instrument and registration number. Adverse events will be described by duration (start and end dates and times), severity, outcome, treatment, and relationship to study drug or, if unrelated, possible cause.

Prior to performing any study-related activities, written informed consent, and authorization must be signed and dated by the patient. If the patient is not literate, the reading of the term will be done by a data collector or the principal researcher, and then the signature will be taken by request. Consent must be obtained prior to performing any study-related collection activities.

After collecting, analyzing data and writing technical documents, the results will be disclosed to patients and professionals who were involved in this study. In addition, the data will be provided to the Brazilian Ministry of Health for future investments in this area.

#### 4.5.1 Cinnamon preparation

To produce the cinnamon to be encapsulated will be use the stick, processed in powder form and the final product was cinnamon extract 0.1%. To obtain the raw material extraction will be performed with drying was done by spray dryer of *C verum*. In addition to the physical-chemical test performed by the manufacturer, the microbiological test will be performed, with values within the normal range for counting bacteria, fungi, and yeast, and the purity test with the counting of heavy metals, such as lead, copper, and antimony.

Cinnamon has authorized the use in Brazil and is part of the research exempt from authorization. Therefore, the concept of “Access to Genetic Heritage” available in Provisional Measure 2186–16/2001 does not apply. Remember that this spice has its widespread use in tropical regions of the world.

After the acquisition of cinnamon powder by a specialized industry, the weighing, encapsulation, and repetition of the quality control tests, as physicochemical, will be carried out in a handling pharmacy that has the green quality seal, the excellence in franchising and the Sinamm Diplomation. The weighing will be computed on an analytical balance.

#### 4.5.2 Blinding

Due to the objectives of the study, the identity of the test and control treatments will not be known to the principal investigator, data collection team, laboratory analysis laboratory, or patients. The following study procedures will be applied to ensure the triple concealment of study treatments.

(1) Access to the randomization list will be strictly controlled;
(2) The capsules, packaging, and labeling of test and control treatments will be identical to maintain confidentiality about the type of treatment;
(3) The concentrations of cinnamon or placebo in each capsule will be the same to minimize the breach of treatment confidentiality.

The treatment received by each person will be revealed after the completion of the clinical intervention and after the study database has been completed.

During the study, blinding can only be broken in emergencies when knowledge of the patient's treatment group is necessary for patient management. When possible, the investigator should discuss the emergency with the attending physician before revealing which group the patient is in.

Participants will be disengaged from study medication if necessary, for their safety or if the participant needs emergency surgery and information is requested on all treatment interventions. This is expected to occur very rarely, or never. Only in the event of an emergency where the participant cannot be adequately treated without knowing the identity of the study medication will this be revealed.

In an emergency, the primary care physician where the patient is being followed or the treating physician will be informed if the patient is receiving cinnamon or placebo. If there is no problem with this, the study will move on.

If it is really necessary to “break” the blinding, then the study coordinator/supervisor will contact the study team members responsible for the confidentiality of the randomization information to retrieve the sealed document.

A protocol bypass form must be completed for any event that requires unraveling the participant's assignment.

In the event of blinding “breakage”, the following information will be recorded:

(1) The identification of the participant;
(2) The reason for the “break”;
(3) The member of the study responsible for the breakage of blinding;
(4) A list of people who are no longer blind to the treatment of the person concerned.

#### 4.5.3 Monitoring and security

Information on all serious adverse events, adverse events, recruitment, and retention, data integrity and data quality will be available for review by designated research group members. Interim safety analyses will be performed periodically throughout the study. While participant safety is the primary concern of the study, it is difficult to formulate precise discontinuity or interruption guidelines that cover all possible situations that may arise.

Adverse events, particularly severe ones, will need to be carefully considered by the research group regarding treatment group imbalances. There are no safety concerns expected with the drugs administered in this study. Cinnamon has been studied not only by the research group, but by other researchers around the world, so there is no reason to believe that its safety and tolerability are a significant problem. In addition, the group will analyze safety data and ensure the scientific validity and ongoing merit of the study.

#### 4.5.4 Protocol violation

Investigators may implement a deviation or change in protocol to eliminate an immediate risk to study participants without prior approval from the ethics committee. As soon as possible, the deviation or change implemented, the reasons for this and, if appropriate, the change (s) to the proposed protocol (s) will be submitted:

(1) To the ethics committee for review and approval/assent;
(2) To the analysis group with the members of the research group, if necessary;
(3) For the regulatory authority.

#### 4.5.5 Data recording and retention

The data will be manually and electronically recorded by the lead researcher in excel, will be shared at the end of the study with 2 researchers who will assist the statistical analysis and control of the data and/or will be checked by 3 separate researchers 1 will check the data entered manually and 2 others will check the electronic data.

To maintain confidentiality, all laboratory samples, evaluation forms, reports and other records will be identified by a coded number and initials only. All study records will be kept in a locked file and code sheets that link the patient name to a patient identification number will be stored separately in another locked file.

Clinical information will not be disclosed without written permission of the participant, except for scientific disclosure (respecting the confidentiality of information such as the name and address of patients) or when necessary for monitoring by the Brazilian Ministry of Health. The researcher must also comply with all applicable privacy regulations.

The database will be protected from unauthorized access by established security procedures (login and password), and appropriate backup copies of the database and files involved in the study will be made. The database will be stored on the main researcher's computer along with any updates. At critical situations of the protocol (eg, interim reporting and final reporting), data for analysis is blocked and cleared according to established procedures. All study documents (patient records, informed consent, etc) will be kept protected for 5 years after the end of the investigation. To ensure data is stored, copies of archives will be archived.

### 4.6 Statistical analysis

All variables will be analyzed descriptively, with numerical and visual summaries. The 2 groups will be compared to check for differences in socioeconomic and clinical variables between the 2 groups at baseline. Numerical variables will be analyzed using the *t* test and categorical variables will be analyzed using the chi-square test.

For HbA1c analysis (in%), the change in value from baseline to end of the study will be considered as the primary endpoint and the means of both groups will be compared using the *t* test for independent groups. The significance level for this test will be 0.05. The analysis will follow the intention-to-treat principle, that is, the participant is analyzed in the group to which he was assigned, regardless of completing treatment as prescribed.

Secondary analyses will include means tests (*t* test) for numerical secondary variables, proportional tests for binary secondary variables, and chi-square test for categorical variables with more than 2 categories. Although we continue to use the significance level of 0.05, these tests will be interpreted with caution as the study was not designed to have statistical power for all of these tests. These tests will be considered exploratory.

Depending on the outcome of the primary outcome, exploratory analyzes will incorporate socioeconomic and clinical variables using regression analysis, where the final HbA1c value at the end of the study is the response variable, and the initial value, socioeconomic, and clinical variables, and the group of treatment will be the explanatory variables. Before statistical analysis, we will evaluate the amount and type of missing values. If necessary and possible, we will use multiple imputations to analyze the data. However, we will also perform the analysis with the full data and the final reports will contain the various analyzes so that the reader can compare them (sensitivity analysis).

Statistical analyses will be performed on SPSS version 25 (SPSS Inc., Chicago, IL) and Stata/SE 15.0 (Stata Corporation, College Station, TX) software for data analysis. Graphics will be done in R Studio.

## 5 Discussion

This study describes the protocol for a randomized controlled trial to evaluate the efficacy of *C verum* in reducing glycemia and lipids in people with T2DM. Facilities in using herbal medicines, especially cinnamon in this population, include feasibility for acquisition, application, and low cost. In addition, the product can easily be added to the daily lives of people with the disease.

The main contribution of this clinical trial is the development of an intervention that may be adjuvant to the current treatment already prescribed in health facilities in Brazil and around the world, allowing patients to insert a herbal product into their daily lives. In the region where the study will be conducted, the northeast region of the country, the population lacks economic resources and does not have sufficient access and coverage to health, as advocated by the World Health Organization. This study will be the first to investigate cinnamon to reduce glycemic, lipid and anthropometric levels in Brazil. In case of favorable results, this therapy may be used as an alternative or additional medicine in cases where only oral antidiabetic agents are used. In addition, nurses in their clinical practice, and by improving their activities, can promote the use of the product to minimize future complications of patients with diabetes and people who do not have the disease.

## Author contributions

**Conceptualization:** José Cláudio Garcia Lira Neto, Marta Maria Coelho Damasceno.

**Data curation:** José Cláudio Garcia Lira Neto, Marta Maria Coelho Damasceno.

**Formal analysis:** José Cláudio Garcia Lira Neto, Márcia Aparecida Ciol.

**Funding acquisition:** Marta Maria Coelho Damasceno.

**Investigation:** José Cláudio Garcia Lira Neto.

**Methodology:** José Cláudio Garcia Lira Neto, Roberto Wagner Júnior Freire de Freitas, Márcio Flávio Moura de Araújo.

**Project administration:** Marta Maria Coelho Damasceno.

**Resources:** Marta Maria Coelho Damasceno.

**Software:** José Cláudio Garcia Lira Neto, Márcia Aparecida Ciol.

**Supervision:** Marta Maria Coelho Damasceno.

**Validation:** José Cláudio Garcia Lira Neto, Marta Maria Coelho Damasceno, Márcia Aparecida Ciol, Roberto Wagner Júnior Freire de Freitas, Márcio Flávio Moura de Araújo.

**Visualization:** José Cláudio Garcia Lira Neto.

**Writing – original draft:** José Cláudio Garcia Lira Neto.

**Writing – review and editing:** José Cláudio Garcia Lira Neto, Carla Regina de Souza Teixeira, Gerdane Celene Nunes Carvalho, Kenya Waléria de Siqueira Coelho Lisboa, Danilo Ferreira de Souza, Jéssica de Menezes Nogueira, Regina Lúcia Lino Marques, Ana Maria Parente Garcia Alencar.

José Cláudio Garcia Lira Neto orcid: 0000-0003-2777-1406.
